# AI-driven breathomics for early detection of CFTR modulator resistance in cystic fibrosis

**DOI:** 10.1097/MS9.0000000000005085

**Published:** 2026-04-30

**Authors:** Muhammad Hamid Khan, Ali Umair Tariq, Waqar Ahmed Cheema, Mahnoor Fatima

**Affiliations:** a Department of Medicine, King Edward Medical University, Lahore, Pakistan; b Department of Medicine, Gujranwala Medical College, Gujranwala, Pakistan; c Department of Medicine, Independent Medical College, Faisalabad, Pakistan; d Department of Medicine, Shaheed Ziaur Rahman Medical College, Rajshahi Medical University, Bogura Bangladesh

*To the Editor,*

Cystic fibrosis (CF) is an autosomal recessive disorder most prevalent in the Caucasian population, caused by a defective cystic fibrosis transmembrane conductance regulator (CFTR) gene, inherited from both parents. The CFTR gene controls ion transport across epithelial surfaces. When mutated, it produces an absent or dysfunctional protein, disrupting chloride movement, and reducing water transport, leading to thick secretions in multiple organs[1].

CFTR modulators (the CFTR protein enhancement drugs ivacaftor, tezacaftor, and elexacaftor) work to treat the base CFTR mutation through their effect on CFTR protein activity. The triple combination elexacaftor/tezacaftor/ivacaftor (ETI) received FDA approval in 2019 to treat patients who possess at least one F508del allele, which includes approximately 90% of the U.S. CF population. Some patients, particularly those with rare or non-F508del CFTR variants, face treatment limitations because they do not qualify for these therapies or experience no treatment benefits, or their symptoms return, or they achieve insufficient long-term results[2]. The ability to track patient progress requires different approaches because rare variants that do not respond to modulators and acquired resistance cases, which include loss of initial response over time, both create monitoring challenges.

Breathomics is the comprehensive analysis of exhaled breath volatile organic compounds (VOCs). It has applications in patients with obstructive lung disease, such as asthma and chronic obstructive pulmonary disease, as it provides opportunities for biomarker discovery, for which traditional statistical methods often cannot effectively handle complex breath data. These VOCs serve as indicators for disease detection, treatment follow-up, and prognosis assessment. Artificial intelligence (AI) and machine learning (ML) can detect hidden VOC patterns, improving accuracy and ensuring instantaneous diagnosis. In CF, VOC profiles distinguish patients from healthy individuals, as FeNO studies show a temporary or persistent increase in fractional exhaled nitric oxide after therapy[3]. Breath profiles show rapid changes that occur within weeks after ETI starts, while these changes link to lung function clinical improvements according to emerging evidence. AI-based breathomics technology provides a promising method for non-invasive early assessment of CFTR modulator treatment response in CF patients. The system also has the potential to detect treatment resistance in patients who are at high risk of developing suboptimal treatment outcomes. The system needs more research to establish its ability to detect specific treatment resistance patterns[4].

Recent medical literature has shown that breath analysis using exhaled breath condensate (EBC) can detect worsening of disease before the development of clinical symptoms in CFTR modulator-resistant variants. Zang *et al* performed metabolic profiling by UPLC-MS in 210 EBC samples collected from CF patients before, during, and after acute pulmonary exacerbations (APE) and under stable conditions. Data demonstrated that classification between pre-APE and stable CF samples was possible with good sensitivities (85.7% and 89.5%), specificities (88.4% and 84.1%), and accuracies (87.7% and 85.7%) for the pediatric and adult populations, respectively[5]. In another clinical study with a data set of 42 888 breath signal entries, researchers developed eNose prototypes for the detection of biomarkers, such as ammonia, ethanol, and acetone, and then applied an XGBoost machine learning classifier for multiclass disease variant prediction. Selvaraj *et al* have found that machine learning (ML) can detect the disease variants in human exhaled breath biomarkers with 98.36% overall classification accuracy in multiclass disease prediction and classification accuracy of 99.77, 94.91, and 96.56% toward ammonia, ethanol, and acetone, respectively[6]. The studies show potential for using ML-based breath analysis in cystic fibrosis and related fields, but do not study CFTR modulator resistance directly. The research results authenticate wider disease monitoring applications, including tracking disease progression and treatment effects of modulators.

The analysis of EBC breathomics through ML-based analysis demonstrates potential for tracking CFTR modulator treatment. The first problem exists because EBC collection and analysis methods have not achieved standardization, which makes it difficult to compare results from different research facilities[7]. Secondly, ML models need extensive datasets while the available data about rare variants and resistance cases remains insufficient for testing purposes. The third hindrance is that most clinicians lack sufficient understanding of AI tools, which prevents them from using these technologies. Lastly, developing regions face equipment accessibility challenges because of expensive analytical devices, challenging the application of advanced technologies[8].

The current situation requires new monitoring methods that can track patients who experience symptom relapses while using CFTR modulators for their rare variants and possible resistance. Machine learning combined with EBC breathomics enables medical professionals to detect treatment response and new health problems through non-invasive methods at earlier stages. This research requires standardization to establish common EBC collection methods and analysis procedures. Research partnerships at multiple centers should focus on gathering data from cohorts with rare variants and modulator treatment. Governments and health systems should provide more equipment so organizations can implement this method, which requires specialized tools outside of dedicated facilities.

## Data Availability

Not applicable to this study.
